# Differences in allergic symptoms after the consumption of egg yolk and egg white

**DOI:** 10.1186/s13223-021-00599-2

**Published:** 2021-09-25

**Authors:** Kei Uneoka, Satoshi Horino, Ayafumi Ozaki, Haruka Aki, Masako Toda, Katsushi Miura

**Affiliations:** 1 Department of Allergy, Miyagi Children’s Hospital, 4-3-17 Ochiai, Aoba-ku, Sendai, 989-3126 Japan; 2 Laboratory of Food and Biomolecular Science, Graduate School of Agricultural Science, Tohoku University, Sendai, Japan

**Keywords:** Allergens, Egg hypersensitivity, Egg yolk, Egg white, Dietary egg proteins

## Abstract

Hen’s eggs are one of the most common causes of food allergy. Although hen’s eggs are known to cause more gastrointestinal symptoms than other foods, it is not known whether there is a difference in organ-specific symptoms between egg yolk (EY) and egg white (EW). The present study aimed to determine whether there are organ-specific differences in the immediate symptoms of EY and EW in patients with hen’s egg allergies. We retrospectively investigated the immediate symptoms and treatment contents of those who had a positive result in an oral food challenge (OFC) of boiled whole EY or 10 g of boiled EW in our hospital from January 2013 to July 2019. We compared 80 patients in the EY-OFC-positive group with 106 patients in the EW-OFC-positive group. The EY-OFC-positive group had significantly fewer respiratory symptoms and significantly more gastrointestinal symptoms than the EW-OFC-positive group and had significantly more gastrointestinal symptoms only. In terms of treatment, significantly fewer patients in the EY-OFC-positive group required beta 2-agonist inhalation, and a significantly higher proportion of patients did not require treatment. Compared to EW, EY is more likely to cause gastrointestinal symptoms and less likely to cause respiratory symptoms. It may be necessary to discriminate between EY and EW allergy during diagnosis.

## Introduction

Intake of hen’s eggs (HE) is a common cause of food allergy in childhood [1]. Heated egg yolk (EY) contains less protein than egg white (EW) and is relatively less allergenic [2]. Most patients with HE allergy can safely consume heated EY. However, some patients exhibit immediate allergic symptoms [2]. The oral food challenge (OFC) is the gold standard diagnostic method to assess food allergy and can reproduce food allergen-induced symptoms [3]. Furthermore, complications can occur in various organs depending on the type of food consumed [4]. For example, allergic reactions to HE are more likely to co-exist with gastrointestinal symptoms than allergic reactions to cow’s milk, soybeans, or wheat [4]. However, it remains unknown whether differences exist between symptoms induced by ingesting heated EY and EW in patients with HE allergy. Based on the allergenic differences between heated EY and EW, we hypothesized that there would be differences in organ-specific symptoms based on the ingestion of boiled EY and EW.

## Materials and methods

We retrospectively analyzed the clinical backgrounds, organ-specific symptoms, and treatments in the OFC-positive groups ingesting boiled EY and EW. This study was approved by the Ethics Committee of the Miyagi Children’s Hospital (approval no. 478). We obtained written informed consent from all participants and parents before performing the OFC.

We collected the data of patients who had positive OFC results due to ingesting boiled whole EY (approximately 16 g) or 10 g of boiled EW at Miyagi Children’s Hospital from January 2013 to July 2019. OFC was recommended in patients with a history of HE allergy or positive IgE (sIgE) specific to EY or EW. OFC was performed after obtaining informed consent from the participants. Patients were defined as having a history of allergy to HE if they presented with any of the following symptoms on ingestion or contact with HE: skin-mucosal symptoms (e.g., rash, swelling, pruritus, flushing, edema), respiratory symptoms (e.g., pharyngeal discomfort, nasal discharge, sneezing, coughing, wheezing, hoarseness, respiratory distress), gastrointestinal symptoms (e.g., itchy mouth, swollen lips, nausea, vomiting, diarrhea, abdominal pain), cardiovascular symptoms (e.g., hypotension), and neurological symptoms (e.g., decreased vitality, impaired consciousness). Patients with missing data or those undergoing oral immunotherapy for HE were excluded from the study.

All OFCs were performed in the hospital using an open method. EY and EW were strictly separated immediately after boiling at 100 °C for 20 min. Positive OFC was defined as severe allergic reactions of grade 2 or higher according to the anaphylaxis grading score of the 2014 Japanese Guideline for Food Allergy (Table 1) [5].

**Table 1:** Classification of anaphylaxis

Grade	Skin	Digestive organ	Respiratory organ	Circulatory organ	Nerve
1	< Localized > pruritus, erythema, urticaria, angioedema	Oral itch and/or discomfort, Swelling of lips	Pharyngeal pruritus and/or discomfort	–	–
2	< Systemic > pruritus, erythema, urticaria, angioedema	Nausea, 1–2 occurrences of vomiting and/or diarrhea, Transient abdominal pain	Slight nasal congestion and/or rhinorrhea, Sneezing once or twice, Sporadic coughing	–	Decrease in activity level
3	Above symptoms	Repeated vomiting, and/or diarrhea, Persistent abdominal Pain	Marked nasal congestion and/or rhinorrhea, Repeated sneezing, Persistent cough, Laryngeal pruritus	Tachycardia (increased ≥ 15 beats/min)	Sense of unease
4	Above symptoms	Above symptoms	Laryngeal tightness, Wheezing, Dyspnea, Hoarseness, Barking cough, Cyanosis, Dysphagia	Arrhythmia, Decreased blood pressure	Unrest, fear of death
5	Above symptoms	Above symptoms	Respiratory arrest	Severe bradycardia, Marked decrease in blood pressure, Cardiac arrest	Unconsciousness

Not all symptoms are essential. The symptom grade was determined according to the highest grade organ symptom. Grade 1 was not regarded as anaphylaxis

Patient data, including sex, age, allergic complications, allergic history of HE, total IgE and sIgE levels (to EY, EW, and ovomucoid), immediate symptoms during OFC, and treatment details, were evaluated. Serum total IgE and sIgE levels were measured within 6 months before OFC. EY or EW was administered in three divided doses (1/10–3/10–6/10) at 30-min intervals. Stepwise OFC was performed in the following order: boiled whole EY, 1 g boiled EW, 10 g boiled EW, and boiled whole egg. EY-OFC-positive patients were not challenged to the EW-OFC. When patients accidentally ingested a tolerated amount of EY or EW and had no allergic symptoms, the lower steps of OFC were skipped. When adverse reactions occurred, patients were closely monitored. Beta 2-agonist inhalation was carried out for intermittent coughing and wheezing. Intramuscular administration of adrenaline was indicated for patients with prolonged grade 4 or higher symptoms.

Statistical analyses were performed using JMP 13 (SAS Institute Inc., Cary, NC, USA) with Fisher’s exact and Wilcoxon rank-sum tests. We considered *P* < 0.05 as indicating statistical significance. For sIgE levels, values < 0.34 U_A_/mL and > 100 U_A_/mL were approximated to 0.34 and 100, respectively.

## Results

**Fig. 1:**
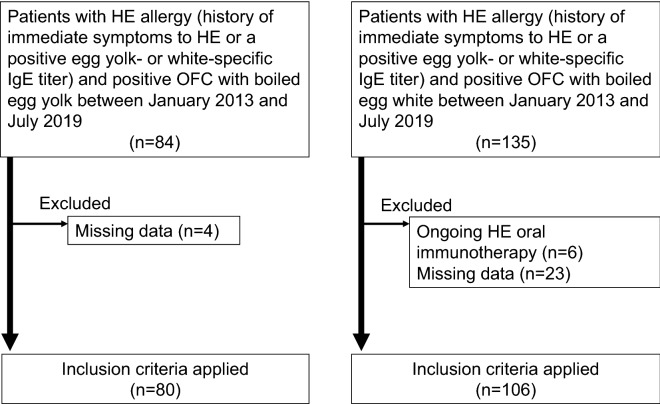
Schematic representation of the study participants’ dispositions. *HE *hen’s egg, *OFC* oral food challenge

We screened 84 and 135 patients with positive OFC to boiled EY (the EY-OFC-positive group) and boiled EW (the EW-OFC-positive group), respectively; finally, data from 80 and 106 patients, respectively, were analyzed (Fig. 1). The EY-OFC-positive group had a significantly lower number of patients with a history of HE ingestion than did the EW-OFC-positive group (61.3% vs. 99.1%) (*P* < 0.001). Serum total IgE (394.5 vs. 242.5 IU/mL) (*P* = 0.007) and sIgE (to EY [8.0 vs. 2.6 U_A_/mL] [*P* < 0.001], EW [37.6 vs. 11.8 U_A_/mL] [*P* < 0.001], and ovomucoid [24.2 vs. 6.6 U_A_/mL] [*P* < 0.001]) levels were significantly higher in the EY-OFC-positive group (Table 2).

**Table 2:** Characteristics of participants

	Positive OFC with boiled egg yolk	Positive OFC with boiled egg white	*P*-value
Number	80	106	
Male (%)	59 (73.8%)	72 (67.9%)	0.421
Age (years)	3.3 (1.7–4.9)	3.5 (2.1–5.1)	0.359
Atopic dermatitis (%)	49 (61.3%)	66 (62.2%)	1.000
Bronchial asthma (%)	24 (30.3%)	31 (29.2%)	1.000
History of egg ingestion (%)	49 (61.3%)	105(99.1%)	< 0.001
Immediate egg allergy history (%)	51 (63.8%)	76 (71.7%)	0.270
Anaphylactic egg allergy history (%)	10 (12.5%)	24 (22.6%)	0.087
Total IgE (IU/mL)	394.5 (175.5–1279.5)	242.5 (105.0–640.5)	0.007
Egg yolk-specific IgE (U_A_/mL)	8.0 (3.9–15.7)	2.6 (0.9–7.9)	< 0.001
Egg white-specific IgE (U_A_/mL)	37.6 (17.9–74.1)	11.8 (5.9–31.9)	< 0.001
Ovomucoid-specific IgE (U_A_/mL)	24.2 (9.2–47.2)	6.6 (1.9–19.2)	< 0.001

Data are presented as the median (interquartile range) or number (%). Statistical analyses were performed using Wilcoxon rank sum test or Fisher’s exact test. Serum levels of egg yolk-, egg white-, and ovomucoid-specific IgE were measured by ImmunoCAP assay (Thermo Fisher Scientific, Uppsala, Sweden)

*IgE* immunoglobulin E, *OFC* oral food challenge

The EY-OFC-positive group also had significantly more gastrointestinal symptoms (70.0% vs. 55.7%) (*P* = 0.049), especially diarrhea (22. 5% vs. 11.3%) (*P* = 0.046), and significantly fewer respiratory symptoms (22.5% vs. 37.8%) (*P* = 0.037) than the EW-OFC-positive group (Table 3).

**Table 3:** Symptoms, anaphylaxis severity scores, and treatments administered to participants with positive oral food challenge results

	Positive OFC with boiled egg yolk	Positive OFC with boiled egg white	*P*-value
Number	80	106	
Symptoms			
Skin (%)	44 (55.0%)	73 (68.9%)	0.066
Respiratory (%)	18 (22.5%)	40 (37.8%)	0.037
Cardiovascular (%)	0 (0.0%)	0 (0.0%)	1.000
Neurologic (%)	3 (3.8%)	8 (7.5%)	0.356
Gastrointestinal (%)	56 (70.0%)	59 (55.7%)	0.049
Abdominal pain (%)	30 (37.5%)	34 (32.1%)	0.533
Nausea or vomiting (%)	27 (33.8%)	31 (29.2%)	0.527
Diarrhea (%)	18 (22.5%)	12 (11.3%)	0.046
Gastrointestinal only (%)	29 (36.3%)	22 (20.8%)	0.021
Anaphylaxis (%)	4 (5.0%)	10 (9.4%)	0.401
Anaphylaxis severity score			
Grade 2 (%)	53 (66.3%)	60 (56.6%)	0.225
Grade 3 (%)	22(27.5%)	33 (31.1%)	0.629
Grade 4 (%)	5 (6.3%)	13 (12.3%)	0.214
Grade 5 (%)	0 (0.0%)	0 (0.0%)	1.000
Treatment			
Antihistamines (%)	45 (56.3%)	73 (68.9%)	0.091
Inhalation of β_2_ stimulant (%)	8 (10.0%)	27 (25.5%)	0.008
Transfusion (%)	2 (2.5%)	0 (0.0%)	0.184
Steroid (%)	4 (5.0%)	2 (1.9%)	0.405
Adrenaline (%)	1 (1.3%)	0 (0.0%)	0.430
No treatment (%)	28 (35.0%)	22 (20.8%)	0.044

Data are presented as number (%)

*OFC* oral food challenge

A higher proportion of patients in the EY-OFC-positive group had only gastrointestinal symptoms (36.3% vs. 20.8%) (*P* = 0.021) than did those in the EW-OFC-positive group. The EY-OFC-positive group had a lower proportion of patients treated with beta 2-agonist inhalation (10.0% vs. 25.5%) (*P* = 0.008) and a significantly higher proportion of patients without medical treatment (35.0% vs. 20.8%) (*P* = 0.044) than the EW-OFC-positive group. In the EY-OFC-positive group, patients with only gastrointestinal symptoms (n = 29) were significantly older than patients without gastrointestinal symptoms (n = 24) (4.9 vs. 2.4 years, respectively) (*P* = 0.001). However, there was no significant difference in the total IgE and sIgE levels (to EY, EW, and ovomucoid) between the two groups. In the EY-OFC-positive group (n = 29), the median time for gastrointestinal symptoms to appear from the first intake of EY was 120 min. None of the EY-OFC-positive groups met the diagnostic criteria of the international consensus guidelines for the diagnosis and management of food protein-induced enterocolitis syndrome (FPIES) [6].

## Discussion

The major allergens of EW include ovomucoid and ovalbumin. Ovomucoid is known to be heat resistant. On the contrary, ovalbumin is known to be sensitive to thermal denaturation, and thus its allergenicity is decreased by heating. Furthermore, an in vitro study on the allergenic changes of EW proteins compared fried EW, boiled EW (at 100 °C for 10 or 30 min), and baked EW at 170 °C [7]. The study showed that neither the temperature nor the duration of heat treatment reduced the antigenicity of ovomucoid [7]. However, they reported that IgE reactivity to ovalbumin was at its lowest when EW was boiled for 30 min, and that allergenicity was also reduced [7]. In our study, we boiled HE at 100 °C for 20 min. Similar to the previous report, the allergenicity of EW was likely decreased [7]. In preparing EY-OFC, EY were separated from EW immediately after boiling to minimize contamination by EW proteins. The amount of HE proteins present in boiled whole EY was minimal [2]. Therefore, allergens of EW might have had little effect on the reactivity of EY in the EY-OFC-positive group.

The proportion of patients with gastrointestinal symptoms among OFC-positive patients was higher for whole eggs than for other foods [4, 8]. In this study, the EY-OFC-positive group had significantly more gastrointestinal symptoms than the EW-OFC-positive group. The results of previous studies, as well as our findings, suggest that whole eggs are more likely to cause gastrointestinal symptoms than other foods, and consuming EY is more likely to cause gastrointestinal symptoms than consuming EW.

We previously reported that EY-OFC-positive patients had delayed HE tolerance acquisition, and it may be possible to estimate HE allergy prognosis by administering EY-OFC [9]. The major allergens in IgE-mediated EY allergy are thought to be chicken serum albumin (Gal d 5) and YGP42 (Gal d 6) [10]. However, it remains unclear whether it is these major EY allergens or other EY components that provoke gastrointestinal symptoms in EY-OFC-positive patients. To the best of our knowledge, this is the first study to report the differences in clinical symptoms induced by boiled EY and EW in patients with HE allergy. Our results may be useful for predicting the appearance of organ-specific symptoms when EY-OFC shows a positive result.

This study had some limitations. First, psychogenic reactions might affect subjective symptoms when using the open OFC method. Second, the retrospective nature of the study may have led to selection bias.

In conclusion, this study found that boiled EY was more likely to provoke gastrointestinal than respiratory symptoms compared to boiled EW in patients with HE allergy. Furthermore, HE allergy may include EY allergy and EW allergy separately.

## Data Availability

Not applicable.

## Abbreviations

HE: Hen’s egg
EY: Egg yolk
EW: Egg white
OFC: Oral food challenge
sIgE: Specific IgE
FPIES: Food protein-induced enterocolitis syndrome
Gal d 5: Chicken serum albumin
Gal d 6: YGP42
